# Burden of Treatment among Elderly Patients with Cancer: A Scoping Review

**DOI:** 10.3390/healthcare9050612

**Published:** 2021-05-19

**Authors:** Adem Sav, Sara S. McMillan, Adeola Akosile

**Affiliations:** 1 School of Public Health and Social Work, Faculty of Health, Queensland University of Technology, Brisbane 4059, Australia; adeola.akosile@gmail.com; 2 School of Pharmacy and Medical Sciences, Griffith Health, Griffith University, Southport 4215, Australia; s.mcmillan@griffith.edu.au

**Keywords:** treatment burden, elderly cancer patients, financial burden, scoping review

## Abstract

Background: The objective of this scoping review is to understand how treatment burden is experienced in elderly patients with cancer and what the most prevalent dimensions of treatment burden are among this population. According to one conceptual model, there are six dimensions of treatment burden, including financial, medication, administrative, time/travel, lifestyle, and healthcare. Methods: A scoping review methodology framework was used to collected data from EMBASE CINAHL (Cumulative Index to Nursing and Allied Health Literature), Medline/PubMed, Scopus, Web of Science, Embase, and Cochrane from 2000 to March 2020. Studies which focused on treatment burden among elderly patients with cancer (+65 years) were selected. Data were extracted using a standardized proforma. Results: The results identified 3319 total papers. Of these, 24 met the inclusion criteria and were included in the scoping review. A significant proportion of these studies was conducted in the United States (*n* = 10) using self-reported, cross-sectional data. Financial burden was the most prevalent dimension of treatment burden, with 11 studies focusing on the direct and indirect costs associated with cancer treatment. Other but less obvious aspects of treatment burden elderly patients experienced included the length of time taken to access and administer treatment and medication-related burdens. Conclusions: Emerging findings suggest that the financial aspects of cancer treatment are a significant burden for most elderly cancer patients. Personalized healthcare interventions targeting ways to reduce and screen for treatment burden, particularly those related to cost, are urgently needed.

## 1. Introduction

As one of the leading causes of mortality worldwide, cancer remains a topic of significant focus for healthcare professionals and researchers [1]. The health-related quality of life in people living with cancer can be significantly diminished because of the nature of the disease and associated treatments [2]. There is widespread evidence of the invasive nature of cancer treatment and the detrimental impact on a person’s social, financial, and psychological wellbeing [2,3,4]. Indeed, people receiving cancer treatment usually experience a range of adverse events [5], and while there have been considerable advances in cancer treatment [6], such developments can result in additional burden for the patient. For example, shortened hospital stays and treatment with oral oncolytics rather than intravenous chemotherapy involve work related to the administration of oral agents, implementation of dosing strategies, and observing for adverse events [4].

It is also common for people who experience cancer to have other co-morbidities, such as diabetes, arthritis, cardiovascular disease, asthma, and mental illness [7]. This is particularly relevant to the ageing population, with 70% of all patients with cancer in the United States of America (USA) expected to be 65 years or older by 2030 [8]. In Australia, the Cancer Council estimates one in two men and one in three women will be diagnosed with cancer by the age of 85 [9]. Worldwide, there is an 11-fold increased incidence of malignancies in persons aged 65 years and older compared to younger adults [10]. This is of particular concern for this population, considering other risk factors, such as limited endurance and poor physical functioning [4]. Experiencing and managing co-morbidities whilst simultaneously undergoing invasive cancer treatment can also lead to treatment burden. 

Treatment burden has been defined as the work or workload/tasks patients need to do and their impact on patients’ wellbeing [11,12,13,14,15]. In broad terms, treatment burden is concerned with the negative experiences a person can go through whilst undergoing treatment [16]. Although treatment burden is often inseparable from disease burden, it is not based on the natural history of the disease, but on the need to treat the disease [16]. Gallacher et al. [12] explored the experience of treatment burden and identified four key dimensions that contributed to the sense of burden: learning about treatments and their consequences, engaging with others and mobilizing support, adhering to treatment and lifestyle changes, and monitoring treatments. A review conducted by Sav et al. [16] suggested that various factors can contribute to treatment burden, such as a person’s age, their medical history, and associated treatment. The authors also suggested that treatment burden can be dynamic and change during the course of a person’s illness [16]. For example, the financial burden experienced by patients can change due to medical treatment or hospitalization because of symptom flare-ups. More recently, Deman et al. [17] drew attention to the sociological aspect of treatment burden, suggesting that burden is not only brought about by the workload associated with a treatment, but also the impact of that workload on everyday activities and a person’s identity. For example, compared to young and working adults, the authors suggested that older persons (who are likely to be retired) may be more likely to accept treatment burden, which they believe is brought on by ageing and living with comorbidities [17].

Although treatment burden is a significant issue for people undergoing cancer treatment, there is limited knowledge about the severity of treatment burden amongst the elderly population and which of its dimensions occur more often and under which circumstances. This information gap has contributed to our inability to measure its impact or identify ageing population most at risk, thereby affecting the role of health professionals in alleviating the associated treatment burden for this population. Hence, a crucial first step in understanding the severity of treatment burden amongst elderly patients with cancer is to collate literature surrounding treatment burden among this population.

**Figure 1:**
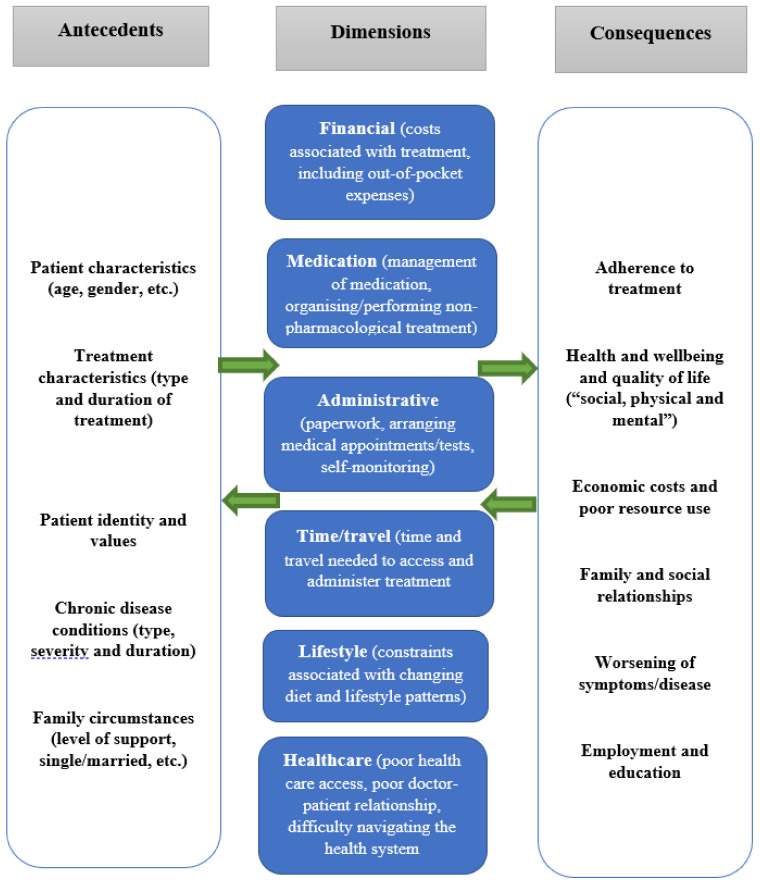
Conceptual framework of treatment burden (based on Sav et al., 2017).

The aim of this scoping review is to understand how treatment burden is experienced by elderly people with cancer and what the most prevalent dimensions of treatment burden are among this population. Data collection and thematic synthesis in this scoping review are guided by a conceptual framework of treatment burden [16]. This framework suggests that there are six commonly accepted dimensions of treatment burden, including financial, medication, administrative, time/travel, lifestyle, and healthcare (see Figure 1). Other potential dimensions of treatment burden (that do not fit the conceptual framework) are also included in the collection and thematic synthesis. Although we use systematic methods to gather data, our aim is not to assess the quality of the selected literature. Rather, given that high levels of treatment burden can lead to treatment non-adherence, treatment adverse events, poor quality of life, and ineffective use of finite health resources [11], we want to “scope” the literature and provide information on strategies for health professionals to further assist this vulnerable population.

## 2. Materials and Methods

A scoping review methodology framework was used because it identifies key concepts, research gaps, and evidence to inform practice, policymaking, and research [18,19].

Database search and review of abstracts:

- The review was guided by the steps outlined by Arksey and O’Malley’s [20] methodological framework for conducting scoping studies;
- Classifying the research question: The research questions of this scoping review were: (a) how is treatment burden experienced in elderly patients with cancer? and (b) what are the most prevalent dimensions of treatment burden among this population?
- Finding the relevant studies/search strategy: Databases searched included EMBASE CINAHL (Cumulative Index to Nursing and Allied Health Literature), Medline/PubMed, Scopus, Web of Science, Embase and Cochrane. We searched for terms relating to the population (elderly patients), the scope (cancer), and the subject (treatment burden) and combined these. Terms included: aged OR elder* aged hospital patient OR frail elderly OR institutionalized elderly OR very elderly OR older; AND geriatric* cancer* OR advanced cancer OR neoplasms* OR malignant* OR chemotherapy* OR radiotherapy* OR drug therapy; AND “treatment burden*” OR “burden of treatment*” OR “medication burden*” OR “burden of medication*” OR “financial burden*” OR “economic burden*” OR “time burden*” OR “workload burden*” OR “cost burden*” OR “cost of illness*” OR “family burden*.” The authors worked with a librarian to conduct the literature search;
- Choosing the studies: Studies published in English between 2000 and 2020, which focused on treatment burden (i.e., dimensions of treatment burden as outlined in Figure 1) were included in the search, which was conducted in March 2020. Because of the focus on elderly patients, only studies involving at least 50% of participants over the age of 65 were included. This resulted in many papers that focused on treatment burden (which should otherwise be included in a review of treatment burden), being excluded because they did not focus on people over the age of 65 years [21,22]. The original authors of several papers were contacted for confirmation regarding the age distribution of their sample if unreported. Similarly, only articles in which cancer was the dominant illness (i.e., 50% or more of the study sample had a cancer diagnosis) were included in the analysis. To capture a wide range of literature, studies were included if they reported primary data from elderly patients (65 years or older) regardless of sample size, study design, method of data collection, cancer type (including site or stage of disease), and treatment (i.e., surgery, immunotherapy, chemotherapy, etc.). Exclusion criteria (see Table 1 below) included studies that focused on caregivers; children or adolescents; non-research articles, i.e., reviews, letters, review articles, case studies, and opinions; and research that is published in languages other than English. Studies which focused on cancer survivorship were only included if they contained information regarding treatment burden. Economic evaluations or studies that did not directly assess the level of treatment burden from patients but instead used a registry or database to calculate the level of treatment burden (i.e., financial burden based on the number of medications purchased by a patient within a particular timeframe) were also excluded. Previous research has shown that treatment burden is a subjective concept and one person’s response may be very different from that of another person undergoing the same treatment [16]. Hence, we believed that registries or databases that determine the level of treatment burden based on the number of medications a person is taking (e.g., more than five prescription medications) do not completely assess treatment burden among patients. Lastly, studies addressing treatment burden at the societal level (not individual), i.e., global economic burden of disease, were excluded.

### Patient and Public Involvement

We did not involve patients or their families in the conduct of this scoping review. However, the keywords used to search the literature and the conceptual framework used in the study were informed by our previous research and consultations with people experiencing chronic illness.

## 3. Results

### 3.1. Description of Studies

**Figure 2:**
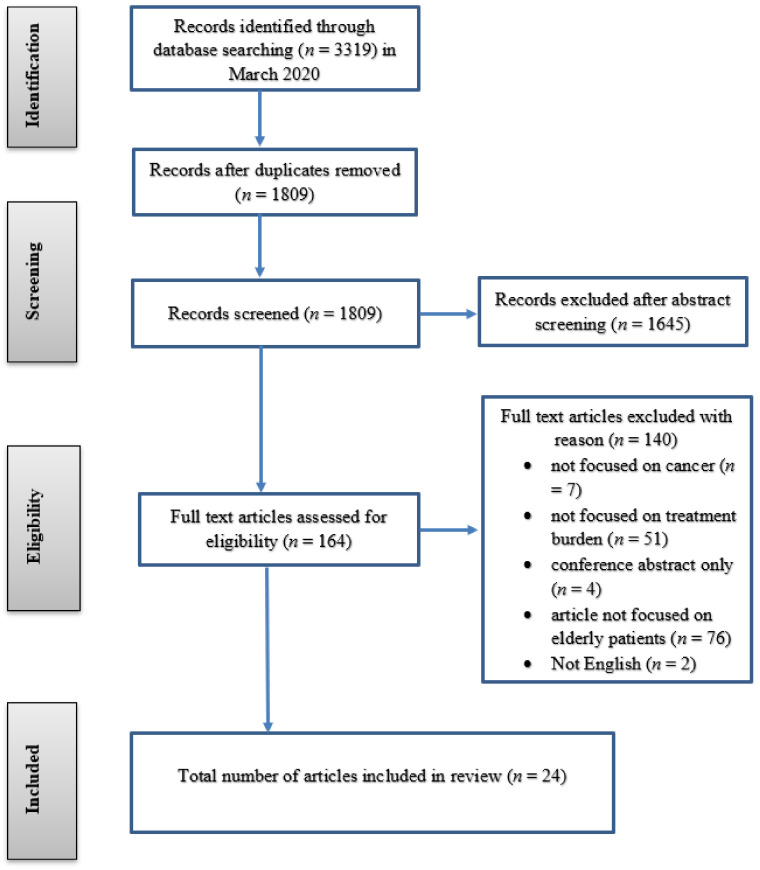
Inclusion flowchart.

A total of 3319 abstracts were identified; 1510 duplicates were removed, and 1809 abstracts were reviewed by two independent authors (AS and SM) for relevance with respect to the inclusion/exclusion criteria. Following initial exclusion, the full-text articles of 164 papers were assessed for eligibility by the same two authors. Reference lists of each article were also screened to locate relevant articles missed by the initial database search; an additional four articles were identified and included. A total of 24 papers were included in the review (see Figure 2). Data were extracted by the lead author using a thematic synthesis proforma including, year of publication, authors, country, data collection, results/outcomes, and study strengths/limitations (see Supplementary Material). Using the standardized proforma, each study was synthesized thematically to generate descriptive and analytical themes/dimensions [23], in accordance with the dimensions outlined in the conceptual framework. However, other areas of interest relating to treatment burden (that do not fit the conceptual framework) were also analyzed from each included paper.

Of the 24 included studies, a significant proportion were conducted in the USA (*n =* 10) [24,25,26,27,28,29,30,31,32,33]. The remaining studies were from other developed countries, including three from the Republic of Ireland [34,35,36], and Australia [37,38,39], two each from the United Kingdom [40,41] and Republic of Korea [42,43], and one each from Canada [44], Iran [45], Finland [46], and Scotland [47]. Most studies used quantitative survey methods (*n =* 17 out of 24). 

The studies had varying sampling sizes, ranging from 6 [41,47] to 8931 participants [32]. Most studies used self-reported, cross-sectional data, although 3 papers had a longitudinal research design [24,25,26]. Overall, 16 papers focused on any form of cancer (i.e., colorectal, stomach, breast, lung, liver, or kidney cancer), 4 on prostate, 2 on colorectal, and 2 on breast cancer. Four studies focused on cancer survivors [24,31,32,35].

The experience of treatment burden for elderly patients with cancer was conceptualized using the dimensions in the conceptual framework for the study. However, other areas of interest relating to treatment burden (that do not fit the conceptual framework) were also analyzed. The data extracted from studies revealed the following three dimensions of treatment burden in order of prevalence: (i) financial burden, i.e., out-of-pocket expenses and disruption to employment and income, (ii) treatment burden related to time, and (iii) medication burden. 

### 3.2. Financial Burden

Financial burden was the most prevalent dimension to emerge, with almost half of all studies (*n =* 11) focusing on the financial and out-of-pocket costs (OOPCs) associated with cancer treatment. Direct expenses, such as the cost of prescription medication, healthcare specialist fees, travel costs to and from the hospital for treatment, diagnostic or imaging fees, and other hidden costs, such as absenteeism and loss of employment, were problematic [26,27,31,32,33,34,35,37,43,45,46].

For direct expenses, patients found themselves paying substantial amounts for outpatient appointments, medical equipment and medication, and medical tests or procedures [37]. In Australia, Gordon et al. [37] found that 50% of men diagnosed with prostate cancer reported an OOPC of AUD 8000 (approximately EUR 5100), while 75% of men spent up to AUD 17,000 (approximately EUR 11,000) for mostly specialist fees, hospital services, medical equipment, and medication. Mojahedian et al. [45] found that the cost of radiotherapy plus surgery in patients in Iran with local nonmetastatic disease formed the greatest share of medical costs, followed by radiotherapy plus hormone therapy. In Finland, the highest mean OOPCs over a six-month period among patients with cancer were for medication (EUR 110) [46]. Aiming to measure the OOPCs borne by survivors of colorectal cancer from the point of initial diagnosis to completion of initial follow-up, Ó Céilleachair et al. [35] found that 90% of survivors in Ireland reported some OOPCs, with an average incurred cost of EUR 1589 (SD = EUR 3827, median EUR 638, interquartile range EUR 100–€1450). Financial burden of cancer treatment was further emphasized by Zafar et al. [33], who described the experiences of patients with various types of cancer requesting copayment assistance in the USA. The authors found an average OOPC of USD 432 (approximately EUR 357) per month for those over 65 years of age undergoing cancer treatment. Both copayment applicants (*n =* 190) and non-applicants (*n =* 64) were equally likely to sell possessions, work or have family members work longer hours, and use their savings to help to cope with the financial burden of cancer treatment [33]. In a study conducted in the Republic of Korea, Min et al. [43] focused on people living with colorectal, stomach, lung, liver, or kidney cancer. Most participants (*n =* 2366; 87.6%) had experienced financial burden that was more than moderate; 39.2% (*n =* 1059) had received financial help or a loan, and 17.8% (*n =* 481) had sold property to afford cancer treatment [43]. Although there was limited information on the extent of financial burden from various demographic backgrounds, there was some information to suggest higher levels of financial burden among racial or ethnic minorities, mainly in the USA (Hispanic and African American participants) [27,31].

An aspect that intensified financial burden was regular medical appointments and testing. For example, one participant in Ó Céilleachair et al.’s [34] study reported attending 8 specialist appointments within the previous 12 months, with a total cost of EUR 960 (although time needed for these visits was not reported). Although private health insurance subsidized EUR 640 of the total cost, the rest remained an OOPC to the patient [34]. Furthermore, this amount excluded associated transportation costs to access treatment and increased utility bills because of the greater time at home [34].

There was variability in OOPCs associated with treatment, time, stage, or severity of the cancer [26]. Ó Céilleachair et al. [35] and Mojahedian et al. [45] both found that patients at the late stage of colorectal cancer reported higher OOPCs. Gordon et al. [37] in Australia found that higher expenses were identified amongst participants who were recently diagnosed with prostate cancer. Having private health insurance did not seem to exempt persons with cancer from financial burden; the addition of co-payments for specialist and hospital fees resulted in treatment discontinuation [37]. Participants with private health insurance reported double the OOPCs than those participants without insurance, regardless of time since cancer diagnosis [37].

It was common for some participants to delay recommended treatment, consider alternative treatment options, or even forgo treatment altogether because of high OOPCs. Gordon et al. [37] found that just under 10% of patients (*n =* 289) decided against treatment due to the associated cost. A study by Zheng et al. [32] in the USA identified that nearly a quarter of cancer survivors over >65 years asked for lower cost medication, with some participants choosing to skip medication doses, take less medication, and delay filling prescriptions [32]. In Min et al.’s [43] study, which examined the factors influencing financial burden, just over 10% (*n =* 276) of participants had changed or stopped treatment due to financial burden. Finally, a qualitative phenomenological study in Australia found that the lack of awareness of services (relating to financial subsidies) and costs prevented participants from accessing financial assistance, leading to treatment non-adherence [39].

Participants reported indirect effects of their cancer treatment, such as changes in employment, including reduction in hours or early retirement (before the official retirement age of 67 in Australia) [37], increased utility bills, particularly for the telephone and heating, and needing new clothes because of weight loss attributable to treatment side-effects [34]. The ability of a patient to be able to pay for their cancer treatment depended on both their income and personal circumstances [35]. Fenlon et al. [40] reported decreased income, with participants in the UK having to borrow money even after therapy completion. For individuals not working at the time of diagnosis, the financial and economic impact of cancer was even more prominent [34]. 

Patients aged 65 years and over used several strategies to cope with the financial burden related to cancer treatment. One common approach was to reduce the money spent on leisure and basic needs, such as clothing and food [33]. Some participants in Tran et al.’s study admitted selling possessions or property [48]. Gordon et al. [37] found that 38% (*n =* 289) of participants withdrew on total savings, 22% increased their credit card limit, and 8% sold assets to enable them to accommodate extra treatment expenses. Reliance on family and friends for emotional and financial support was also common [33,34,42]. 

### 3.3. Time/Travel Burden

Another dimension of treatment burden (see Figure 1) that emerged was time, although there was less information on this compared to financial burden. Meehan et al. [28] analyzed resource use and time utilized during the treatment process at three oncology clinics in the USA. Participants spent a substantial amount of time at the clinic for the management of anemia associated with treatment. For example, the total mean travel and appointment time per patient (defined as the time spent travelling to and from the clinic, waiting before treatment, and the time for injection administration) was 82.7 minutes [28]. These findings correlate with a time and motion study; Richhariya et al. [30] determined the mean time associated with IV Zolendronic acid (ZA) administration in patients with bone metastasis from prostate (65.0 mins; SD 32.5) or breast cancer (72.1 mins; SD 47.1). A similar study by Xie et al. [44] estimated the time spent administrating ZA and a similar medication, pamidronate. The total estimated time patients spent in the clinic (including wait-time) was 56.6 minutes, although the frequency of clinic visits was not reported. When exploring the lived experiences of people living with incurable cancer, Loughran et al. [41] found that the burden of medical appointments, treatments, and procedures left little time for rest or activity, and getting to hospital was difficult for people with limited transport options. Ervin et al. found that the need to travel for treatment for those people living in rural areas added to financial hardship [38].

### 3.4. Medication Burden 

There was limited information in the included studies on medication burden. Some studies reported long-term physical effects post-cancer treatment, such as poor cosmetic results, reduced range of movement of the affected site, fatigue, diarrhea and vomiting, and difficulty in performing daily activities and attending to personal care needs [24,29,34,36,40,41,47]. Azuero et al. [24] examined associations between co-morbidity and predictors of health status among breast cancer survivors. They found that patients reported several adverse events of cancer treatment, including pain, depression, anxiety, and hot flashes, effects which some participants experienced two years post-treatment [24].

A qualitative study by Walker and Andrew [47] on the patient’s experience after photodynamic cancer treatment in Scotland found that five out of six participants experienced photosensitive reactions. Participants also expressed concern for the perceived added burden imposed on their families, with one participated stating: “I used to go to the shops, but I have been unable to because of the lights. Ever since the treatment, my wife has been doing most of the driving” [47] (p. 83). Diefenbach and Mohamed [25] conducted a longitudinal descriptive analysis on regret of treatment decision and its association with disease-specific quality of life following prostate cancer treatment. Although there were low levels of treatment regret, significant and positive associations were found between regret, activity limitation, and bother with sexual and urinary dysfunction. Finally, participants in Loughran et al. [41] reported that treatment side effects caused physical problems, which could fluctuate, causing difficulties. Losing one’s own physical strength and sexual function because of treatment had a substantial psychological effect on participants as it challenged their understanding of the male role and image [28].

## 4. Discussion

The effect of treatment burden on elderly patients undergoing or at the completion of cancer treatment is irrefutable, of which financial burden seems to be the most prevalent and concerning aspect. The reviewed studies indicate that there are numerous direct costs attributable to cancer treatment, such as medications, specialist appointments and consultations, hospital or nursing home care, and special medical equipment, which place a major burden on elderly cancer patients. The financial burden of cancer treatment also extends to other hidden or indirect costs, for example, because of time lost from employment because of treatment or early retirement. Our findings align with other research in this area, clearly stressing the problematic nature of OOPCs and the “financial toxicity” of cancer treatment [33,49,50,51,52]. Financial burden is particularly problematic considering that elderly patients are typically low-income earners (either retired or working part-time) and are more likely to have co-morbidities compared to their younger counterparts. 

Although one could argue that the financial burden imposed on elderly patients undergoing cancer treatment would differ by country and health system, our review suggests this may not be entirely correct. For example, research from the USA, Australia, Scotland, Iran, Finland, and South Korea highlighted the financial toxicity associated with cancer treatment. While the level and impact of financial burden on people may be different in such counties because of varying healthcare systems and cultural beliefs, it is apparent that a substantial proportion of elderly people struggle with the financial demands imposed on them by cancer treatment. Private health insurance coverage, despite being available for some patients, does not appear to be enough to minimize the associated OOPCs. This is because private health coverage is often capped at a particular threshold, and once this is reached, people face an accumulation of direct OOPCs for medical treatment, which even can result in bankruptcy for some [48]. Although we did not set out to identify if people are better off with or without insurance, our findings raise some concerns about the value of insurance in a population with limited financial capacity.

Our research on treatment burden suggests that the financial toxicity experienced by patients with cancer is not confined to this disease but is at the core of a person’s experience of treatment for chronic health conditions in general [16,53]. Evidence has shown that for participants with various chronic health conditions, financial burden is indeed a problematic dimension of treatment burden [14,53]. Financial burden has a detrimental impact on a person’s use of medication and is exacerbated by other types of burden, such as access to healthcare services and the time and travel associated with treatment [53]. It seems that these findings are also common among elderly people undergoing cancer treatment. What is vague in this review, however, but clearly established in prior research [14], is the negative consequences of financial burden on general health and wellbeing. There was very little information about the commonly accepted consequences of financial burden associated with cancer treatment on other issues, such as medication or treatment adherence, disease relapse, leisure activities, and finance for other basic living necessities [49]. Further attention by health professionals and policy makers on the spillover effects of financial burden among elderly patients receiving cancer treatment is required.

While there was evidence of time-related burden in this review, there appeared to be more tolerance of this type of burden when compared with financial burden. The time required to administer treatment and experience of poor health in general may be more socially sanctioned in older people and may be perceived as having less impact on lifestyle when compared to younger people. Indeed, elderly patients with cancer may perceive what they must do and see it as less burdensome or have more time to undertake treatment-related tasks. Alternatively, time burden associated with cancer treatment may also be more socially acceptable as cancer is generally seen as a more serious and life-threatening illness compared to more commonly experienced chronic conditions, such as diabetes or arthritis. Ultimately, the reasons time burden associated with cancer treatment and management is not seen as burdensome as financial cost among elderly people needs further unpacking. 

The above could also be said in relation to medication burden and adverse events of cancer treatment. Although there was some information regarding the burden associated with medication use in our review, this was minimal compared to associated costs. Given the well-reported invasive and burdensome nature of cancer treatment options, we were expecting to find much more information on this issue in our review. Again, this may reflect the tolerance of treatment-related adverse events of this age group. We believe that other commonly experienced adverse events of chemotherapy and radiotherapy may be more tolerated in the elderly than in the younger population. Alternatively, it may be that elderly patients were not able to clearly recognize the adverse events of their cancer because of associated co-morbidities. Although comparing the treatment burden experiences of younger and older patients was beyond the scope of this review, this is clearly an important avenue for future research.

### 4.1. Implications

Our review indicates that the costs associated with cancer treatment for the elderly remain a significant problem worldwide. Oncologists and other leading health professionals have been very vocal about the issue of cancer treatment affordability [51]. Further efforts are needed to alleviate some of the financial burden experienced by elderly patients, and a range of strategies have been offered in the literature: subsidized and/or cheaper medications, discussing cost issues with patients, screening for risk of financial toxicity, referral to financial support services, improved health insurance design, co-payments and financial counselling programs, or workshops focused on financial literacy [48,49,51,54]. Although these options are worthwhile strategies, we believe that patient-centered treatment methods are vital. Notwithstanding the objective or direct financial burden of cancer treatment, we join Carrera et al.’s [51] notion of seeing financial burden of cancer treatment within the wider context of the patient’s individual circumstances. This can be achieved by routinely including cost and affordability discussions as part of patient care alongside information about their personal circumstances (e.g., age, health insurance details, employment situation). This will facilitate discussions about treatment options, side-effects, issues of access and travel, thereby helping to alleviate other aspects of treatment burden. 

Screening for financial burden using short and validated screening tools can identify how patients may be experiencing burden and that this information could be used to triage patients to appropriate support staff and financial resources [49]. Screening tools could assist with overcoming communication challenges (e.g., discomfort in discussing care costs with patients and/or insufficient time to engage in such discussions), which can impact the patient–physician relationship [54]. It is important to ensure that family members are also given an opportunity to become part of cost discussions, as treatment decisions by patients can be made with family members because the economic impact of cancer affects the entire family unit.

Although health professionals have a key role in reducing the risk of financial toxicity among elderly cancer patients, they should not feel alone in this challenge. The issue of financial toxicity will require a collaborative effort from stakeholders, including employers, politicians and policy makers, managers/administrators, community champions and advocates, not-for-profit organizations, and researchers. Only then can evidence-based and patient-centered methods for alleviating financial and treatment burden for cancer care among elderly patients be achieved. Additionally, there is a need to develop interventions that aim to reduce the burden of treatment in such populations. Solely documenting the levels of treatment burden among elderly cancer patients is not enough; it is imperative that those individuals who feel overwhelmed by their treatment be identified and assisted by health professionals and health services within a health system that supports this.

Finally, although only three dimensions of treatment burden were identified in this review, the importance of other dimensions cannot be excluded because of the nature of the included studies. Many of the included studies focused on one or two dimensions of treatment burden (e.g., financial and medication), generally at the expense of the others. Hence, it was difficult to truly understand how treatment burden was experienced by elderly people with cancer. Furthermore, given that a significant body of this research was conducted in the USA (*n =* 10 in our study), we need to think more broadly about the experiences of treatment burden in different health systems and countries. For example, significant differences in financial burden experiences may exist in countries where there is free universal healthcare compared to those which are subsidized with occurring OOPCs. It would be interesting to find out if the hidden costs of cancer treatment, including early retirement from employment and loss of income, would continue to be prevalent among elderly patients in countries where there are no OOPCs for healthcare. There is clearly a need to focus more holistically on treatment burden among different settings for health professionals and policy makers to comprehensively understand its impact upon this vulnerable population.

### 4.2. Limitations

It is possible that we may have missed papers because of our specific decision to only include studies where 50% or more of the sample size comprised participants 65 years and over. This was required to ensure that the findings were indeed focused on elderly cancer patients. However, for some papers, it was difficult to ascertain the distribution of the age of the sample. Hence, we contacted the original authors of such papers for confirmation and read the full paper to determine eligibility. However, we did not always receive a response, and consequently, these papers (without an author’s response) were not included in this review. We also tried to be inclusive of elderly patients in our data search by using elderly-related terms (e.g., above 75 or 80 years of age). However, we do acknowledge that because of the nature of the papers included in the review, the manuscript did not really include many studies that focused on the very elderly. Secondly, although we did not find sufficient information on the other dimensions of treatment burden (e.g., healthcare, lifestyle), this does not mean that elderly patients with cancer do not experience these forms of burden. Rather, it may be because the studies included in this review mainly focused on the financial aspects of treatment burden. Additionally, because our review was restricted to English language only papers, we may have missed key papers in non-English language. Finally, it is worth highlighting that cancer treatment can be very different (e.g., immunotherapy for melanoma compared to stage four chemotherapy for pancreatic cancer). Although we did focus on several types of cancers, future research needs to broaden this scope and assess the levels of treatment burden for each type of cancer. Despite these limitations, this review provides an important snapshot of the published literature and highlights the impact of financial burden of cancer treatment on elderly people.

## 5. Conclusions

Cancer treatment is an invasive and burdensome experience for elderly patients, and much of this burden revolves around the issue of cost and financial toxicity of treatment. While there have seen significant advances in cancer treatment, these advances seem to have done little to alleviate the associated financial toxicity. Further attention on the impact of financial burden of treatment on other aspects of a person’s life and a need for health professionals to screen for this burden is urgently needed.

## Figures and Tables

**Table 1:** Eligibility criteria for article selection inclusion criteria.

**Inclusion criteria**	**All studies contributing to information of treatment burden (in accordance with Figure 1) among elderly patients with cancer.**
	All studies that contain primary data (e.g., survey, interview, observation, etc.) from elderly patients (65 years or older) regardless of sample size, study design, method of data collection, cancer type (including site or stage of disease) and treatment (i.e., surgery, immunotherapy, chemotherapy, etc.).
	Studies published in English between 2000 and March 2020.
**Exclusion criteria**	**Did not specifically focus on elderly patients (+65 years). Excluded if more than 50% of the patients were under the age of 65 or if the mean age of the sample was below 65 years of age.**
	Did not contribute to information on treatment burden among elderly patients with cancer.
	Did not specifically focus on cancer (i.e., less than 50% of the study sample did not have a cancer diagnosis).
	Caregivers or unpaid carers of people with cancer (unless they also experienced cancer themselves).
	Economic evaluations or studies that did not directly assess the level of treatment burden from patients but instead used a registry or database to calculate the level of treatment burden.
	Studies addressing treatment burden at the societal level e.g., the financial burden of treatment on the economic productivity of a country
	Reviews, letters and opinions; and studies.
	Not written in English.

## Data Availability

Data sharing is not applicable in this article.

## Supplementary Materials

The following are available online at https://www.mdpi.com/article/10.3390/healthcare9050612/s1, Table S1: Thematic synthesis of articles.
