# Supplementary material for: Burden of Treatment among Elderly Patients with Cancer: A Scoping Review
Source: Healthcare (Basel). 2021 May 19;9(5):612. doi: 10.3390/healthcare9050612 (PMC8160635; doi:10.3390/healthcare9050612)
Supplement: Supplementary file 1 [file healthcare-09-00612-s001.zip › healthcare-1171053-supplementary.pdf]

Supplementary file Table S1. Thematic synthesis of articles

**Author, Year and Country**	**Aim**	**Study Design and Sample**	**Key findings and Conclusions**	**Strengths and Limitations**
Azuero et al. (2014)<br><br>USA	To examine associations between comorbidity and predictors of health status among older rural breast cancer survivors.	Quantitative research design.<br><br>Baseline data gathered from the Rural Breast Cancer Survivors Study.<br><br>A total of 331 rural participants enrolled (55-90 years); data collected at baseline and every three months for a total of 12 months.<br><br>The following variables were examined: (1) health status, (2) co-morbid conditions, (3) sociodemographic and treatment characteristics, and (4) social support.	A mean number of 3.68 prescription categories (range of 0–12) was reported. The most common prescription category (66.2%; n = 219) was anti-hormonal agents for cancer, followed by medication for high blood pressure and high cholesterol. Commonly associated side effects included: pain (19.9%), depression (14.8%), insomnia/sleep (14.2%), anxiety (13.9%) and hot flashes (5.4%).<br><br>Participants reported a decrease in income level, changes in economic lifestyle, and having to borrow money after breast cancer therapy ended.	Longitudinal research design.<br><br>The study was based on participants who had completed cancer treatment.<br><br>Limited generalizability (sample from one USA state).
Cuffe et al. (2020)<br><br>Republic of Ireland	To explore the experiences of patients living with multiple myeloma.	Qualitative research design.<br><br>Patients with diagnosis of multiple myeloma for less than 1 year (n= 6, mean age = 67.5 years)	Participants highlighted the lack of time and<br>opportunity to talk with health professionals and associated communication issues.<br><br>Participants experienced severe side-effects, which impacted on their life.	Small sample size<br><br>Minimal detail on methodology.<br><br>In-depth discussion of the experiences of patients by

			Fatigue, diarrhoea and vomiting were identified as the worst side-effects they experienced. Steroid toxicity, peripheral neuropathy and diarrhoea were reported.	an experienced interviewer is a strength.
Diefenbach & Mohamed (2007)<br><br>USA	To examine decisional regret among prostate cancer patients and its association with disease-specific quality of life (QOL).	Quantitative research design.<br><br>The baseline questionnaire included demographic, medical variables (e.g. Prostate-Specific Antigen levels) and pre-treatment disease-specific QOL measures (e.g. urinary and sexual dysfunction). Disease-specific QOL measures also administered at six and 12-months.<br><br>793 patients completed all measurement points and had prostatectomy, external beam radiation or brachytherapy (average age 65.57 years).	Although levels of decisional regret were low, regret increased significantly between 6 and 12 months after diagnosis. The increase was substantial for patients treated with prostatectomy compared to patients treated with external beam radiation or brachytherapy. Cross-sectional, significant, and positive associations among regret, activity limitation attributed to urinary dysfunction, and bother with sexual and urinary dysfunction emerged.<br><br>Longitudinally, the change in the level of regret was significantly associated with treatment modality and with the change in bother with sexual dysfunction over the first 6 months after diagnosis.	Longitudinal research design.<br><br>Sample consisted mainly of Caucasian participants with an identifiable social support structure (being married).<br><br>Very brief measures used to assess participant burden.
Ervin et al. (2019)<br><br>Australia	To explore unmet supportive care needs of rural men with cancer to inform improvements in service delivery.	Qualitative research design<br><br>Focus group with 10 participants with cancer<br><br>Data were analysed thematically	Men were 70 years of age (range 53–84).<br><br>Financial distress was raised by many participants. Several told stories of seeking help but were frustrated by the process and their inability to change their	Small sample size<br><br>Only one focus group, which does not allow comparison<br><br>Strength: focus on men and an in-depth

			circumstances. The need to travel for treatment added to<br>this financial hardship.<br><br>Participants expressed that they had little knowledge of the treatment they had received, the reoccurrence of illness, or where to access information about their illness and treatment.<br><br>Physical symptoms (e.g., fatigue) related to cancer or treatment was discussed and the impact of this on their daily life and interests.	discussion of the topic of interest
Fenlon et al. (2013)<br><br>UK	To explore older women's experience of living with breast cancer alongside other health conditions, and to identify their information and support needs and preferences.	Qualitative (thematic analysis).<br><br>Breast cancer survivors (aged 70-90).<br><br>Semi-structured qualitative interviews (n=28) explored lived experiences of breast cancer and other health conditions.<br><br>Two 2 focus groups (n=14) explored omen's information and support needs.	Several women suffered long-term physical and emotional problems as a result of their breast cancer treatment: distress from scars and body changes, poor cosmetic results post-surgery which caused some discomfort and affected simple daily living activities, e.g. getting dressed. Bra's and/or prosthesis were difficult to live with, e.g. they were uncomfortable; bras rubbed or needed to be worn tight to hold the prosthesis; prostheses were heavy.<br><br>Older women desired the need to be treated individually and holistically.	In-depth understanding of treatment experiences of diverse women.<br><br>Findings are based on experience of care more than ten years ago and may not reflect current practice.<br><br>No ethnic minority participants were recruited.

Gordon et al. (2015)<br><br>Australia	To understand the extent, nature and variability of the current economic burden of prostate cancer among Australian men.	Quantitative research design.<br><br>An online questionarie comprising of 68 questions over seven domains: medical history, employment, household finances, private health insurance status, QOL, general socio-demographic variables and out-of-pocket costs (OOPC).<br><br>289 men (mean age 65 years) from various prostate cancer support groups.	The mean total OOPCs reported between diagnosis and survey date by all respondents was AU$9205 (SD AU$14 567) with a median $5000 (interquartile range AU$10 000). Expenditure was largely for specialist fees, hospital services, medical equipment and supplies and medicines.<br><br>Respondents with private health insurance reported double the OOPCs (AU$10 052, 95% CI: AU$7849–AU$12 255) than those without insurance (AU$5103, 95% CI: AU$2458–AU$7747) regardless of time since diagnosis. Reported expenses were higher among men recently diagnosed (median $8000) compared with men diagnosed within the last 3 years (median $5500).<br><br>70% had spent more for their cancer treatment than they expected to, 20% said that the cost of treating their prostate cancer had caused them 'a great deal' of distress. Approximately one quarter of men chose an earlier retirement age, and had stopped work, as a result of their diagnosis.	Focus on men with prostate cancer and comorbidity is s strength.<br><br>There was an opportunity for participants to provide open-ended responses in the questionnaire.<br><br>Some missing data (17%).<br><br>The retrospective and cross-sectional nature of the survey left the results open to recall bias.
Jayadevappa et al. (2010)	To analyze OOPC and indirect costs (OPI) for patients newly	Quantitative research design.	At baseline, there was no difference in the proportion of patients reporting OOPCs across the two treatment groups.	Longitudinal research design.

USA	diagnosed with prostate cancer receiving either radical prostatectomy (RP) or external beam radiation therapy (EBRT).	Two-part, 8-item survey completed at enrolment (baseline), 3, 6, 12, and 24 months post-treatment follow-up.<br><br>Only participants receiving EBRT (n=160) were over the age of 65 (participants receiving RP were aged < 65 years).	At all time-points, a higher proportion of EBRT patients reported requiring more time to travel than the RP group.<br><br>The mean medication costs for RP and EBRT group was \$137 versus \$57.2 at 3-month follow-up; \$95 versus \$70 at 6-months; \$190 versus \$280 at 12-months; \$161 versus \$356 at 24-months.<br><br>At 12 months, a higher proportion of RP patients reported missing work (44.5% vs. 16.3%; $P < 0.0001$) and requiring more help from their caregivers (17.1% vs. 5.3%; $P = 0.0061$) compared with EBRT patients. In contrast, at 6, 12, and 24 months, a higher proportion of EBRT patients reported taking more time to do the usual housework than RP patients.<br><br>Total mean OPI costs for RP and EBRT group were \$5576 versus \$2010 at 3-month follow-up; \$1776 versus \$2133 at 6-months; \$757 versus \$774 at 12-months; \$458 versus \$871 at 24-months.	Could not differentiate between specific items of other OPI costs such as vitamin supplements complementary and alternative medicines, home care and others.<br><br>Limited generalizability as participants were recruited from one large urban academic hospital.
Jones et al. (2019)<br><br>USA	To examine how multimorbidity influences the prevalence of financial burden among older	Quantitative research design.<br><br>Data from the National Health and Aging Trends Study was used (65 years	Cancer without comorbidity was not associated with financial burden (medical-related OR: 1.031, 95% CI: 0.802–1.327; overall OR: 0.847, 95% CI: 0.637–1.126).	Sample sizes for some subgroups were relatively small.

	adults with heart disease, diabetes, or cancer.	and over), with oversampling of African Americans.<br><br>Survey questions: medical-related credit card debt (due to medical costs, paying medical bills over time), overall financial burden (financial help from family, credit card debt, help with food, utilities, etc.), health conditions and demographics/insurance characteristics	Cancer with comorbidity was associated with overall financial burden (OR: 1.568, 95% CI: 1.004–2.447) and nearly associated with medical-related financial burden (OR: 1.398, 95% CI: 0.906–2.158).<br><br>Women, Hispanic and African American participants had higher odds of reporting both medical related and overall financial burden than men and white participants (P <0.05) in nearly all analyses. People with higher incomes tended to report less financial burden (P<0.02) but this association was less consistent with medical-related financial burden.	Comorbidity and cancer may have led to financial problems or financial problems may have preceded cancer with comorbidity.<br><br>The financial difficulties and chronic conditions measured were not comprehensive
Koskinen et al. (2019).<br><br>Finland	To examine the relationship between financial difficulties and HRQoL (health related quality of life) among patients with breast, prostate and colorectal cancer in different stages of the disease as well as to calculate the resulting total financial burden	Quantitative research design<br><br>1978 cancer patients with prostate (n=630), breast (n=840), and colorectal cancer (n=508)<br><br>Participants formed five mutually exclusive groups: primary disease (0–6 months from diagnosis), rehabilitation (6–18 months from diagnosis), remission (subsequent years in remission), metastatic	The median participant age was 66 years (range: 26-96 years). Many were retired (63%), 27% were employed and 4% unemployed or not working.<br><br>In the group that reported very much health-related financial difficulties, the majority were men (78.9%), living alone (55.3%) and unemployed (84.2%). Almost half of the patients were in the remission state of the disease (47.4%).<br><br>During the 6-month period, OOPCs were highest in the palliative care group, compared to other groups. The highest	Cross-section and long-term effects of financial difficulties on HRQoL not assessed.<br><br>Strength: Understanding of financial burden among different groups of cancer patients

		disease (active oncological treat ments) and palliative care.<br><br>The data on the length of the hospitalization or visit, procedures and examinations, and patient level costs, including overheads, equipment and medication for inpatient use were collected, along with HRQoL data.	mean OOPC were for medication and the lowest for travel.<br><br>Financial difficulties, age, high total health care costs and OOPCs had the most significant total negative effect on HRQoL among cancer patients.	
Loughran et al. (2019)<br><br>UK	To explore the lived experiences of people living with incurable cancer, the effects on their lives, their views on rehabilitation, and their perceived rehabilitation needs in palliative care setting.	Qualitative, phenomenological study.<br><br>Participants recruited within a specialist community palliative care service in North East England.<br><br>Semi-structured interviews (25-60 min) conducted with 6 participants.	Participants reported that treatment side effects caused physical problems which could fluctuate and build up to increase difficulties. The burden of medical appointments, treatments, and procedures also added to physical difficulties, leaving little time for rest or activity.<br><br>Coping strategies were needed (e.g., sleeping in cars part way through an evening and drinking less alcohol) in order to avoid sedating side effects.<br><br>Losing one's own physical strength and sexual function due to treatment had a large psychological effect. Loss of abilities and physical strength caused	Limited sample size<br><br>A strength is the in-depth understanding and knowledge obtained.

			embarrassment and challenged understanding of male role/image.<br><br>Rehabilitation services were difficult to access, poorly utilised and referrals were sporadic and consequential. Getting to hospitals could be difficult with limited transport	
Meehan et al. (2006)<br><br>USA	To identify the resource use and time commitment associated with treatment of anaemia with erythropoietic therapy, for both haematology/oncology clinics and patients.	Quantitative research design.<br><br>Involved participants (n=124) from three haematology/oncology clinics (mean age 67 years). Majority of study participants were female (58%) and Caucasian (74%).<br><br>The time and the clinical resources required by staff to perform phlebotomy for monitoring purposes, and to prepare and administer the epoetin alfa injections, were recorded at each study location by trained researchers.<br><br>Patients were asked to complete a questionnaire that	Patients were receiving epoetin alfa injections once per week (68% of patients), once every 2 weeks (15%), once every 3 weeks (5%) or three times per week (2%).<br><br>The total mean travel and appointment time per patient, (time spent travelling to and from the clinic, the time spent waiting before treatment and the time for injection administration), was 82.7 minutes. In addition, 9.9% of participants reported that they missed time from work for clinic visits.<br><br>50% of patients were accompanied to their clinic visit by a caregiver; 9% reported that their caregivers missed time from work to accompany them.	One of the few studies to demonstrate that considerable time and resource use are involved in epoetin alfa administration at haematology/oncology clinics.<br><br>Study was based on travel time reported by patients (self-reported bias).<br><br>Caregivers' travel time was estimated by patients.

		captured demographics and the travel time for patients and caregivers.		
Min et al. (2018)<br><br>Republic of Korea	To describe applicants' condition and to investigate factors influencing financial burden.	Quantitative research design.<br><br>Annual satisfaction telephone survey of Financial Aid Program for cancer patient recipients (n= 2,700) Half of participants were aged 65 years and over.<br><br>Squestions : (1) participant demographic, socioeconomic, and disease characteristics (2) experiences associated with financial burden and the actions or measures taken to cope with them.	Most applicants (87.6 %; n=2,366) experienced significant financial burden; 39.2% (n=1,059) received financial assistance; 17.8% (n=481) sold their property for financial reasons. Just over 10% f participants (n=276) had changed or stopped treatment due to the medical cost.<br><br>Although most applicants experienced a financial burden, the cost of cancer care was a major financial burden for males, younger applicants, parents of childhood cancer patients, low-income groups, applicants who did not have private insurance, and applicants with late-stage cancer.<br><br>Existing applicants received financial assistance less frequently than newly registered applicants (OR, 0.38; 95% CI, 0.31 to 0.48) but had a higher property disposal rate (OR, 1.42; 95% CI, 1.07 to 1.87).	Strength is an evaluation of costs after a public financial aid program for cancer patients<br><br>Cross-sectional and self-reported data.<br><br>The exact level of income was not collected.
Mojahedian et al. (2019)<br><br>Iran	To estimate direct and indirect costs of treatment at different	Quantitative research design.<br><br>Cross-sectional study involving 263 patients	Annual direct medical costs increased as the disease progressed. Medical costs were relatively low for patients in early stages of the disease.	Cross-sectional and only few items measuring financial burden among participants.

	stages of prostate cancer in Iran.	diagnosed with prostate cancer who were referred treatment centers in 2016.<br><br>Participants were classified into 5 groups in terms of treatment stages<br><br>Data collected by face-to-face interviews and other medial/financial documents.	The cost of radiotherapy plus surgery (23%) in patients with local nonmetastatic disease formed the greatest share of medical costs, followed by radiotherapy plus hormone therapy (17%).<br><br>In most cases, participants were compelled to use their savings or that of their family members or borrow money to cover medical costs. This problem arose for most people in the more severe stages of the disease.	One of the few studies conducted in Iran.
O Ceillaechair et al. (2012)<br><br>Republic of Ireland	To investigate the broad spectrum of economic and emotional consequences faced by patients with colorectal cancer, the inter-relationships between these and meditating factors.	Qualitative, phenomenological study.<br><br>Participants recruited from six hospitals.<br><br>Face-to-face interviews (60-90 mins) undertaken with 22 patients and six carers (4 spouses and 2 daughters).	Key themes: OOPCs (both medical and non-medical), "making ends meet" (i.e. managing financially), the role of family and friends, services and entitlements, and emotional costs.<br><br>Hospital travel costs were important for all interviewees, especially for chemotherapy and/or radiotherapy treatment as this required more hospital visits. Some experienced difficulties using public transport (e.g. journey length or treatment side-effects).<br><br>Some respondents had increased utility bills (e.g. telephone and heating) following diagnosis. For those not working at diagnosis, the financial and	Offer in-depth understanding of the experiences of participants with colorectal cancer.<br><br>Small sample size could be considered a limitation, as well as the use of cross-sectional, self-reported data.

			economic impact of cancer was even more pronounced; these individuals generally had smaller pre-diagnosis incomes.<br><br>Almost all patients described how treatment-related changes in their bodies (e.g. weight loss, accommodating a stoma) made it necessary for them to buy new clothes. The associated costs ranged from incidental to burdensome (e.g. entirely new wardrobe).<br><br>Accessing health and social services could also cause emotional strain, because of unhelpful or insensitive staff, or due to the specific circumstances or requirements. For example, one patient was, unexpectedly, required to pay for scans before the results would be released.	
O Ceillaechair et al. (2017)<br><br>Republic of Ireland	To measure the OOPCs borne by survivors of colorectal cancer from the point of initial diagnosis to completion of initial follow-up	Quantitative research design.<br><br>Participants (n=497) were asked to provide information on a range of potential OOPCs, including costs of clinician or GP consultations , prescription medications, appliances and treatment-	Overall, having stage III at diagnosis and being older were significant drivers of OOPCs, and several other factors—such as disease site, employment status and having children—were important in subgroups.<br><br>Approximately 90% of survivors reported some OOPC with an average incurred cost of €1589 (*SD* = €3827,	One of the few studies to document the OOPC for colorectal cancer survivors and how these breakdown by phase of care and type of cost.<br><br>Self-reported data.

		related sundries such as travel and subsistence.	median €638, inter-quartile range €100–€1450). Those aged 70+ had a significantly lower mean OOPC than those <70 (€1160 versus €1948) (*F* = 5.27, *p* = 0.0221). Those in employment at diagnosis had a slightly higher OOPC than those who were not (€1963 versus €1367; *F* = 2.81, *p* = 0.0943).<br><br>Stratification by employment status: in those who were not working at diagnosis, stage III disease was a significant predictor of higher costs (€156.35, 95% CI €22.83 to €289.8, *p* = 0.022) as was having rectal cancer (€201.05, 95% CI €62.53–€339.56, *p* = 0.005). In those who were working at diagnosis, survivors >70years old had a lower OOPC (−€25.87, 95%CI −€40.21 to −€11.53, *p* < 0.0001).<br><br>Stratification by age: in those <70 years old, stage III disease was associated with significantly higher OOPCs (€260.28, 95% CI €27.62 to €492.93, *p* = 0.028) whilst those with children experienced lower costs (−€420.03, 95%CI −€752.35 to −€87.72, *p* = 0.013). For those >70 years, being in employment was associated with significantly higher costs	Fewer stage IV patients participated possibly due to their poorer prognosis.

			(€276.52, 95% CI €67.65–€485.39, p = 0.010).	
Pisu et al. (2018)<br><br>USA	To identify factors that have the strongest impact on health related QOL (HRQOL) for older adults with cancer.	Quantitative research design.<br><br>Participants diagnosed with cancer after January 1 2008 responded to a telephone survey (1457 / 1460).<br><br>Majority were female (59.8%), Caucasian (81.4%). Most prevalent cancer types were breast (23.7%) and prostate (13.0%). Age range 65-99 years (mean age 74.2 years).<br><br>HRQOL was measured using the Physical Component Summary (PCS) and Mental Component Summary (MCS) scores of the 12-Item Short Form Survey (SF-12) from the Medical Outcomes Study.	The factors individually found to be strongly associated with the SF-12 PCS score were symptom severity (physical domain; adjusted R2 5 0.34) and physical support needs (social domain; adjusted R2 5 0.16). Fatigue, pain, disturbed sleep, and drowsiness were the symptoms found to be most strongly associated with SF-12 PCS scores (adjusted R2 0.16).<br><br>The factors found to be strongly associated with SF-12 MCS scores were symptom severity (physical domain; adjusted R2 5 0.32) and number of financial hardship events (social domain; adjusted R2 5 0.15). Fatigue, problems remembering things, disturbed sleep, and lack of appetite were the symptoms found to be most strongly associated with SF-12 MCS scores.	Cross-sectional design.<br><br>Sample consisted of participants with high levels of education.<br><br>Other potentially important variables, which may affect HRQOL, were not measured.
Richhariya et al. (2012)<br><br>USA	To delineate the overall time associated with administration of IV Zoledronic Acid (ZA) in patients with bone metastases	Quantitative research design.<br><br>Data collection at seven oncology clinics: observer form, site questionnaire, and a case report form.	The most common comorbid conditions were musculoskeletal (prostate cancer: 40%), cardiovascular and respiratory (prostate cancer: 73%), and endocrine-related (prostate cancer: 27%).	The observers employed for data collection were rigorously trained to ensure high data quality.

	secondary to breast or prostate cancer.	Mean age for breast cancer patients was 60.6 years (SD 12.6) and for prostate cancer patients was 73.9 years (SD 8.5).	Mean ZA administration time: 72.1 mins (SD 47.1) for breast cancer; 65.0 mins (SD 32.5) for prostate cancer; 69.4 mins (SD 41.8) for both groups combined.<br><br>Mean ZA infusion time: 33.9 mins (SD 15.1) for breast cancer; 29.4 mins (SD 12.1) for prostate cancer; 32.2 mins (SD 14) for both cancers combined.<br><br>The mean time spent by healthcare providers for IV infusion of ZA: 16.4 mins (SD 21.0) for registered nurses; 10.7 mins (SD 20.2) for nurse practitioners; 8.1 mins (SD 6.5) for physicians.	Only direct time data in the clinic were collected; indirect time burden on patients in terms of lost work time or time for travel were not captured. Time related to separate visits to the laboratory for serum creatinine testing was not quantified.<br><br>Possible Hawthorne effect: healthcare personnel administering IV ZA were aware that they were being observed and timed (i.e. may have demonstrated increased efficiency in their activities).
Slavova-Azmanova, et al. (2019)<br><br>Australia	To explore, rural and outer metropolitan cancer patients'perceptions of the communication with their cancer specialists, its impact on the quality of the care they receive, and financial burden.	Qualitative, phenomenological study.<br><br>Participants recruited from Western Australia Cancer Registry (n=40).<br><br>Forty semi-structured interviews undertaken with 20 rural and 20 outer metropolitan participants	Participants were concerned about communication practices regarding treatment options and side effects.<br><br>Some identified a reluctance by their specialist to discuss side effects and attributed this to the health providers' lack of awareness, unwillingness to acknowledge that some treatments can cause harm and a lack of personal experience with the treatment.	Underrepresentation of patients with lung cancer.<br><br>Possibility of interviewer bias because the study was a follow up of another study (same interviewer).<br><br>Strength is the diverse range of participants from

		diagnosed with breast (n=11), prostate n=11), colorectal (n =11) and lung (n=7) cancer	Unexpected post-treatment bills surprised participants. The lack of awareness of services and costs prevented participants from accessing financial assistance, which lead to treatment non-adherence and caused unnecessary stress..	both rural and outer metropolitan areas of Australia.<br><br>In-depth exploration of the topic of interest.
Walker and Andrew (2009)<br><br>Scotland	To gain an understanding of patients' lived experience following photodynamic therapy (PDT) for the treatment of advanced cancer.	Qualitative (phenomenological hermeneutic interpretation).<br><br>Purposive sampling: study participants were recruited from the local treatment centre by the consultant or nurse involved in their care.<br><br>Six semi-structured interviews.	Five of the six participants had experienced some degree of photosensitive reaction and for three of them, this was while being in the car.<br><br>Having PDT for advanced cancer could be likened to riding an ‘emotional rollercoaster’, with alternating relief and worry. A variety of coping strategies were evident such as humour, sheer grit and determination and having a positive attitude<br><br>Also apparent was the lack of knowledge about PDT from some healthcare professionals. Perceived concerns for the added burden imposed on their families	In-depth understanding<br><br>Small sample size can be considered a limitation.<br><br>Cross-sectional and self-reported data.
Xie et al. (2014)<br><br>Canada	To estimate the time and labor costs associated with the administration of ZA and pamidronate in cancer patients with	Quantitative (observational time and motion study).<br><br>All observers were trained by watching and timing a patient visit. Primary outcome: time spent on the	The mean time for ZA preparation for the patients with prostate cancer was 2.7 minutes. The mean infusion time for patients receiving ZA was 20.6 minutes. Total clinic time (including wait time: 56.6 mins.	One of the few studies to compare ambulatory infusion devices with regular infusion devices using the time and motion method.

	metastatic bone diseases.	activities associated with ZA and pamidronate disodium administration.<br><br>Convenience sample: n=26 from Sunnybrook and n=11 from Laval. Mean age 66 years. There was n=19 participants with breast cancer and n=18 with prostate cancer.	Including the wait time, the total time spent in the clinic was 30.1 mins if using ambulatory infusion devices and 185.1 mins if using regular infusion devices.	Small sample size.<br><br>Only involved two hospital-based oncology clinic centers.
Yabrof et al. 2016)<br><br>USA	To estimate the prevalence of financial hardship associated with cancer and identify characteristics of cancer survivors associated with financial hardship.	Quantitative research design.<br><br>Self-administered questionnaire based on the Medical Expenditure Panel Survey.<br><br>Material financial hardship was measured by ever (1) borrowing money or going into debt, (2) filing for bankruptcy, (3) being unable to cover one's share of medical care costs, or (4) making other financial sacrifices because of cancer, its treatment, and lasting effects of treatment. Psychological financial	Slightly more than half of cancer survivors were ≥ 65 years of age. Overall, 20.4% (95% CI, 17.7% to 23.4%) of cancer survivors reported having had any material financial hardship associated with cancer, its treatment, or late and lasting effects of treatment. Working-age cancer survivors experienced significant financial and psychological hardship. There was high OOPCs despite insurance.<br><br>The percentage of cancer survivors reporting any material financial hardship was significantly greater for those age 18 to 64 years compared with those ≥ 65 years of age (28.4% *v* 13.8%; $P < .001$).	The findings provide a snapshot of the prevalence of material and psychological financial hardship in US cancer survivors in 2011.<br><br>Cross-sectional and self-reported data.<br><br>Confirmation of diagnosis, information about stage at diagnosis, specific treatment, timing of recurrence, or other clinical characteristics, were not available.

		hardship was measured as ever worrying about paying large medical bills.	Psychological financial hardship was more common among those age 18 to 64 years than among those ≥ 65 years of age (31.9% *v* 14.7%; *P* < .001).<br><br>Among those age ≥ 65 years, 6.6% reported only material hardship; 7.6% reported only psychological hardship; and 7.1% reported both material and psychological hardship.<br><br>More financial hardship amongst racial/ethnic minorities, the poor, uninsured and with public insurance.	
Yoon et al. (2015)<br><br>Korea Republic	To examine the QOL of older adults with cancer and investigate associated factors	Quantitative research design.<br><br>A total of 339 patients aged (>65 years) who were treated for colorectal, stomach, lung, liver or kidney cancer.<br><br>Questionnaire: assessed the QOL of cancer patients, cancer/treatment characteristics, performance status, optimism, cancer-related health worries, and social support.	Optimism was inversely associated with cancer-related health worries. Social supports, friends and medical professionals were positively associated with optimism, but inversely associated with cancer-related health worries.<br><br>Older adults with more advanced cancer stage at diagnosis (b = −3.23) and poorer performance status (b = −9.00) had significantly lower physical function. Older adults with more advanced cancer stage at diagnosis (b = −2.67) and poorer performance status (b = −4.96) had significantly lower social function.<br><br>Nearly 55% experienced changes in work situation; stopped working permanently	Although the sample was concentrated on five major cancer diseases in Korea, a large proportion of those who were selected were patients with colorectal and stomach cancer.<br><br>Cross-sectional data.

			(25.1%), changed working hours (20.4%) and stopped working temporarily (9.7%).<br><br>79.8% experienced financial burden related to medical costs (17.5% had private healthcare insurance).	
Zafar et al. (2013)<br><br>USA	To describe the experiences of insured patients with cancer requesting co-payment assistance and the impact of health care expenses on well-being and treatment.	Quantitative research design.<br><br>Self-reported observational study, which began 1 month after the baseline study.<br><br>Patients estimated their OOPCs.<br><br>A total of 190/254 participants requested co-payment assistance. Only the participants who requested co-payment support (n=190) had a mean age of 65 years or over.	OOPCs averaged $432 per month (p=0.19) for the >65 years age group.<br><br>Applying for co-payment assistance and communicating with physicians about the cost of care were associated with greater subjective financial burden.<br><br>Co-payment assistance applicants were more likely than non-applicants to employ at least one strategy to cope with costs (98% vs. 78%; $p < .001$). Among those who employed at least one strategy, co-payment assistance applicants were more likely to reduce spending on leisure activities (p=0.005), food or clothing (p=0.021), and borrow money or use credit (p=0.001).<br><br>Both co-payment applicants and non-applicants were equally likely to sell possessions, work longer hours, have family members work longer hours, and use their savings. However, co-payment assistance applicants were more likely than nonapplicants to reduce spending on	One of the few studies that moves beyond just assessing the level of financial burden among cancer patients but also understating the impact of this burden.<br><br>The baseline survey response rate was 38%, which introduces the possibility of non-response bias.<br><br>Not all patients completed a cost diary: 63% of respondents completed at least one monthly cost diary and only 13% completed all four diary entries.

			food, clothing, and leisure activities to help pay for care.	
Zheng et al. (2017)<br><br>USA	To compare the changes in prescription drug use for financial reasons between cancer survivors and individuals without a cancer history	Quantitative research design.<br><br>Data set : 2011 to 2014 National Health Interview Survey (cancer survivors; n=8931) to collate health status information, use of health care services and co-morbid conditions.<br><br>Analysis was stratified by age (nonelderly, ages 18–64 years; elderly, ages ≥65 years) and time since diagnosis (recently diagnosed).<br><br>Measures of change in prescription drug use for financial reasons included: 1) skipping medication doses, 2) taking less medicine, 3) delaying filling a prescription, 4) asking a doctor for lower cost medication, 5) buying prescription drugs from another country, and 6) using alternative therapies.	Non-elderly (<65 years) cancer survivors were more likely to report changes in prescription drug use for financial reasons than those without a cancer history<br><br>Among the elderly population, compared with the control group: 1) 22.6% of recently diagnosed cancer survivors reported asking for lower cost medication (excess change, 5% [95% CI, 1%–9.1%]; 2) on average, approximately 4% of previously diagnosed cancer survivors reported skipping medication doses, taking less medicine, and delaying filling a prescription (excess change, $p<0.05$).	The study used a nationally representative sample to estimate patterns of changes in prescription drug use for financial reasons in both the non-elderly and elderly population of cancer survivors.<br><br>The cancer history was self-reported.<br><br>Time since diagnosis was measured by the difference between age at the time of the survey and age at cancer diagnosis, which may have been subject to measurement error.
